# Prognostic value of amphiregulin and epiregulin mRNA expression in metastatic colorectal cancer patients

**DOI:** 10.18632/oncotarget.10151

**Published:** 2016-06-17

**Authors:** Chen Jing, Yang Han Jin, Zhai You, Qian Qiong, Zhou Jun

**Affiliations:** ^1^ Department of Medical Oncology, The First Affiliated Hospital of College of Medicine, Zhejiang University, Hangzhou, Zhejiang, P.R. China; ^2^ Department of Pathology, The First Affiliated Hospital of College of Medicine, Zhejiang University, Hangzhou, Zhejiang, P.R. China; ^3^ Department of Pharmacy, The First Affiliated Hospital of College of Medicine, Zhejiang University, Hangzhou, Zhejiang, P.R. China

**Keywords:** metastatic colorectal cancer, amphiregulin, epiregulin, meta-analysis, prognostic biomarker

## Abstract

Epidermal growth factor receptor (EGFR) and its ligands amphiregulin (AREG) and epiregulin (EREG) play a central role in the development of colorectal cancer, but the prognostic values of AREG and EREG are controversial. We conducted a meta-analysis of studies that investigated AREG and/or EREG mRNA levels in primary tumors to determine their prognostic value in metastatic colorectal cancer (mCRC). In addition, RAS status was assessed. Relevant articles were identified by searching the EMBASE, PubMed, and Cochrane Library databases. Hazard ratios (HR) with 95% confidence intervals (CIs) were calculated using a random-effects model. Nine studies involving 2167 patients were included in this meta-analysis. High AREG expression was associated with longer overall survival (OS) and progression-free survival (PFS). High EREG expression was also associated with prolonged OS and PFS. In RAS wild-type (WT) patients who received anti-EGFR therapy, high AREG and EREG expression was associated with longer OS. Our results indicate that high AREG and EREG mRNA expression are independent favorable prognostic biomarkers in mCRC. The expression of these ligands should be considered when evaluating prognoses in RAS-WT patients receiving anti-EGFR therapy.

## INTRODUCTION

Colorectal cancer is the fourth most common cancer in both sexes and the second leading cause of cancer-related death worldwide, and metastatic colorectal cancer (mCRC) is associated with a poor prognosis [1]. mCRC with wild-type RAS (RAS-WT), which accounts for more than half of cases, is dependent on the epidermal growth factor receptor (EGFR) signal pathway [2]. There is evidence that treatment with anti-EGFR mAbs cetuximab or panitumumab improves the overall survival rate of RAS-WT patients [3–5]. Nevertheless, only two-thirds of RAS-WT patients respond to these therapies, suggesting that other biomarkers besides RAS still need to be researched [6, 7].

Amphiregulin (AREG) and epiregulin (EREG), which are ligands of EGFR, are overexpressed in colorectal cancer at both the mRNA and protein levels [1, 8–11]. Ligand-induced EGFR activation plays a key role in tumor proliferation, invasion, and migration through the RAS-RAF-MAPK and PI3K-AKT-mTOR pathways [12], and ligand binding results in EGFR activation in cell lines [13, 14]. In addition, knock-out of the AREG or EREG genes reduces the therapeutic efficacy of cetuximab [11]. Thus, AREG and EREG might be predictive biomarkers in mCRC. [15]

High ligand mRNA expression typically correlates with favorable outcomes in patients receiving cetuximab or panitumumab treatment [9, 15–24]. Some studies found that high AREG and EREG mRNA levels in tumors act as independent favorable prognostic factors [17, 21], while other studies [18, 22, 23, 25] reported that only EREG expression acts as an independent prognostic marker in mCRC patients18,22,23,25. Variation in study designs and sample sizes may contribute to this discrepancy [26]. Furthermore, most investigations only included patients treated with anti-EGFR, and thus have not been able to differentiate between prognostic and predictive effects. The direct impact of AREG and EREG levels on patient survival remains inconclusive. Here, we conducted a systematic review of the literature and used meta-analysis to investigate the prognostic utility of EGFR ligand expression, including both AREG and EREG, in mCRC patients. The relationship between EGFR ligand expression and RAS state was also evaluated to exclude potential pathway-related interactions.

## RESULTS

### Patient demographics and clinical characteristics

**Figure 1:**
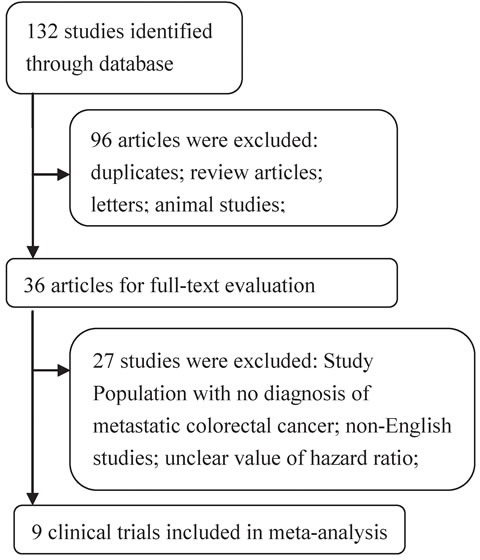
Flow chart depicting the study selection process

Biomarker analyses for 2167 mCRC patients were included in this systematic review of nine published studies, three of which were RCTs (Figure 1 and Table 1). Six of the articles restricted their analyses to the impact of AREG mRNA expression on overall survival (OS), while eight evaluated EREG expression. Seven articles assessed the effects of both AREG and EREG expression on progression-free survival (PFS). Some studies used the minimum *p*-value method to select high and low mRNA expression cutoffs [9, 19, 21, 23, 25, 26], while other studies used receiver operating characteristics (ROC) [17, 22, 24]. Eight studies used anti-EGFR mAb therapy alone or in combination with chemotherapy, and one study used only chemotherapy. Seven studies evaluated cetuximab and one study evaluated panitumumab. Six studies restricted analysis to KRAS-WT tumors, and three studies restricted analysis to KRAS-WT and NRAS-WT tumors. Table 1 summarizes the background therapy details and the *p*-values for high *vs*. low AREG/EREG expression; histopathological details are shown in Supplementary Table 1.

**Table 1:** Summary of studies included in meta-analysis

Author(Publication Date)	Trial phase	Therapy(trial)	ITT	*p* value of HR AREG high *vs* low		*p* value of HR EREG high *vs* low		Jadad score
				OS	PFS	OS	PFS	
Khambata-Ford(2007)	phase 2	cetuximab	110	NR	<0.001	NR	0.0002	3
Jacobs(2009)	phase 2	CT+cetuximab	220	<0.0001	<0.001	<0.0001	<0.001	4
Saridaki(2011)	phase 2	CT+cetuximab	112	0.013	0.018	0.004	0.002	4
Pentheroudakis(2013)	phase 2	CT+cetuximab	226	0.0002	NR	0.0009	NR	3
Strimpakos(2013)	phase 2	CT+cetuximab	222	< .0001	NR	0.006	NR	3
Cushman(2015)	phase 3 RCT	CT±cetuximab(CALGB 80203)	238	0.923	0.144	0.012	0.016	5
Llovet(2015)	phase 2	CT+cetuximab	105	0.05	0.001	0.053	0.09	3
Seligmann(2016)	phase 3 RCT	CT±panitumumab(PICCOLO)	696	0.18	0.50	0.001	0.16	5
Stahler(2016)	phase 3 RCT	CT(FIRE 1)	238	0.11	0.03	<0.001	0.002	4

CT, chemotherapy; ITT, overall intention-to-treat population; HR, hazard ratio; AREG, amphiregulin; ERGE, epiregulin; High, high expression; low, low expression. NR, not reported in the publication

### Effects of AREG and EREG expression on OS in mCRC

**Figure 2:**
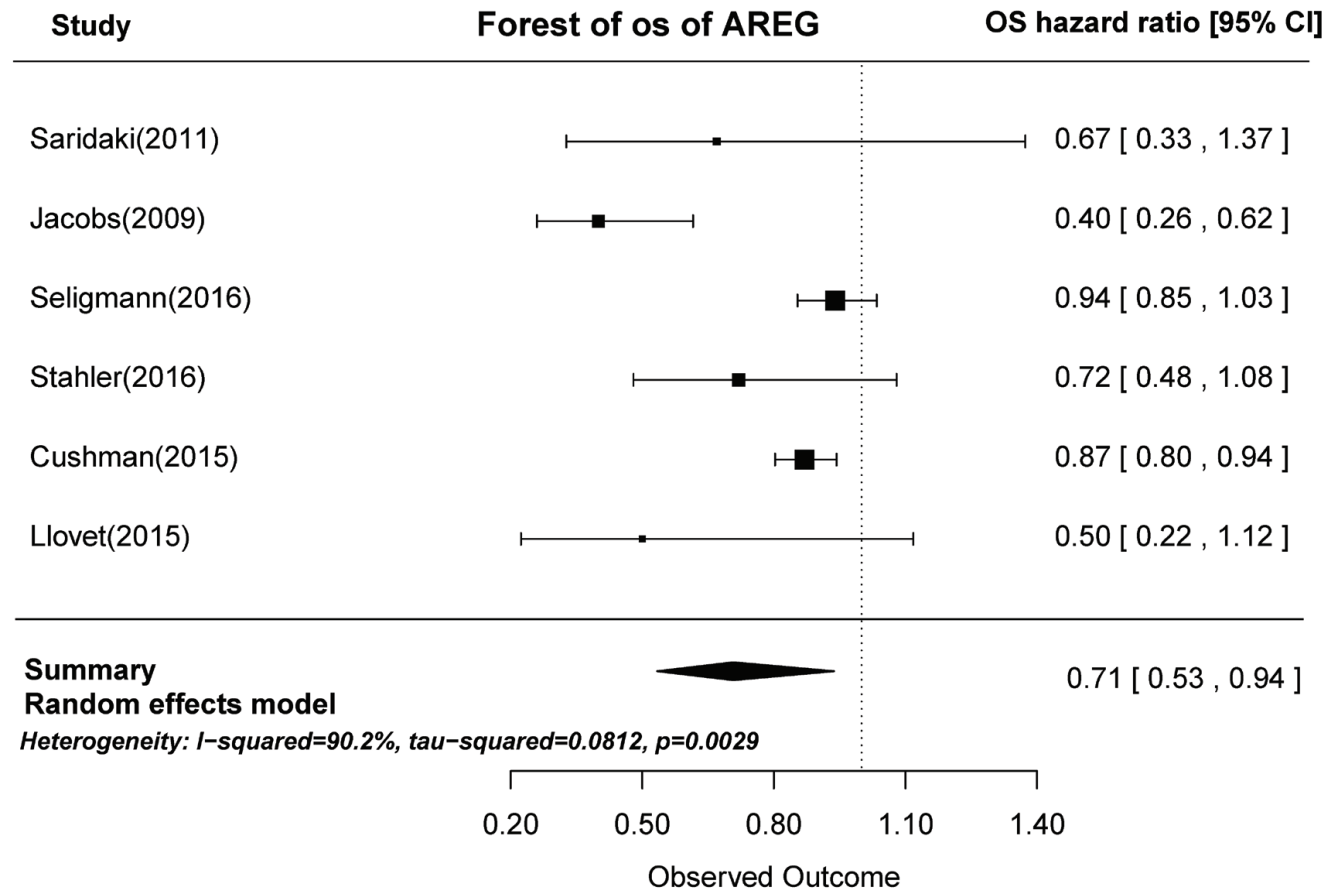
Forest plot of overall survival in high and low tumor AREG mRNA expression subgroups

**Figure 3:**
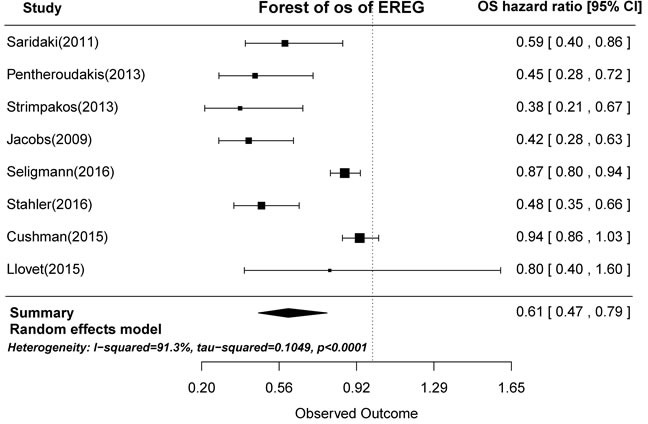
Forest plot of overall survival in high and low tumor EREG mRNA expression subgroups

In prognostic analyses, high AREG and EREG mRNA levels in tumors were associated with prolonged OS. High AREG mRNA expression was associated with longer OS compared to low AREG expression (HR = 0.71, 95% CI: 0.53-0.94, *p* = 0.0029; Figure 2). Similarly, high EREG expression compared to low EREG expression had longer OS. (HR = 0.61, 95% CI: 0.47-0.79, *p* < 0.0001; Figure 3).

### Effects of AREG and EREG expression on PFS in mCRC

**Figure 4:**
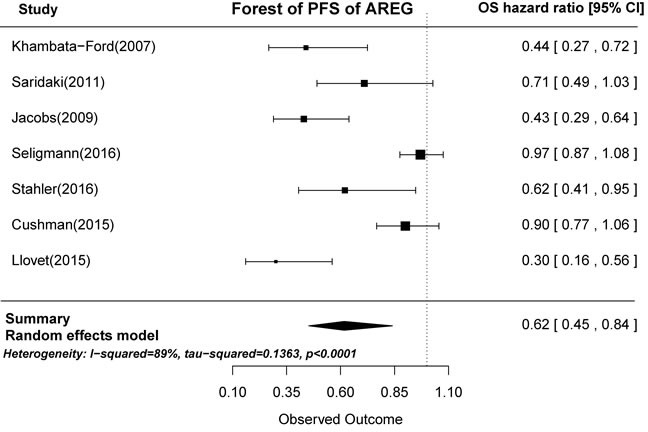
Forest plot of progression-free survival in high and low tumor AREG mRNA expression subgroups

**Figure 5:**
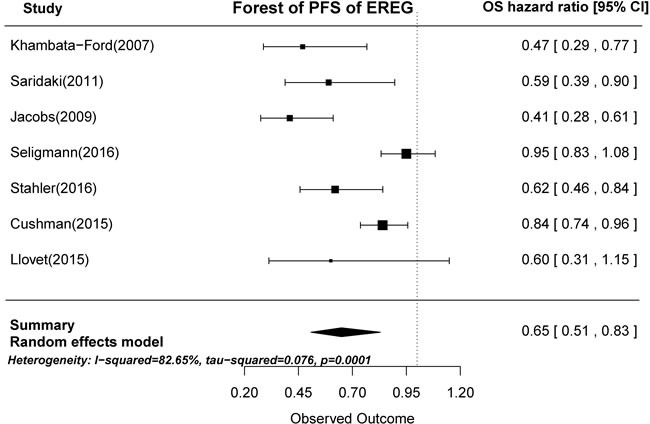
Forest plot of progression-free survival in high and low tumor EREG mRNA expression subgroups

Based on the gene expression results of the seven articles examined, tumors with high AREG expression were associated with longer PFS than those with low AREG expression (HR = 0.62, 95% CI: 0.45-0.84, *p* < 0.0001; Figure 4). Similarly, high EREG expression was associated with longer PFS than low EREG expression (HR = 0.65, 95% CI: 0.51-0.83, *p* = 0.0001; Figure 5).

### Effects of AREG and EREG expression depending on RAS state in mCRC

RAS mutations were detected in 556 of 1553 patients (36.4%). In RAS-WT patients treated with anti-EGFR therapy, high AREG expression was associated with both longer PFS (HR = 0.85, 95% CI: 0.76-0.95, *p* = 0.0005) and longer OS (HR = 0.37, 95% CI; 0.16-0.86; *p* = 0.02). OS, but not PFS (*p* = 0.06), was also longer in patients with high EREG expression compared to those with low EREG expression (HR = 0.54, 95% CI: 0.31-0.940, *p* = 0.03). OS and PFS in patients with RAS-MT were not associated with AREG or EREG expression. These results, shown in Table 2, indicate that AREG and EREG levels should be considered when evaluating the effects of anti-EGFR therapy in RAS-WT mCRC patients.

**Table 2:** Meta-analysis for ligand expression effect of overall survival and progression-free survival in patients with metastatic CRC assigned to RAS state

Subgroup	survival	Gene	*N*	HR(95%CI)	*P*-value	Heterogeneity, tau2; P; I^2^
RAS WT	OS	AREG	4	0.37(0.16-0.86)	0.02	0.65;<0.001;90.19%
		EREG	4	0.54(0.31-0.94)	0.03	0.26; <0.001; 86.31%
	PFS	AREG	3	0.85(0.76-0.95)	0.005	0;0.14;0.02%
		EREG	4	0.72(0.51-1.01)	0.06	0.09;0.0116;88.36%
RAS MT	OS	AREG	2	1.09(0.91-1.36)	0.37	0;0.5458;0%
		EREG	2	1.07(0.9-1.24)	0.4	0;0.9239;0%
	PFS	AREG	1	0.93 (0.77–1.13)	0.46	NA
		EREG	2	0.95(0.82-1.10)	0.48	0;0.9072;0%

WT, wild type; MT, mutation type; OS, overall survival; PFS, progression-free survival; AREG, amphir, egulin; ERGE, epiregulin; N, number of included studies; HR, hazard ratio; NA, not applicable

### Publication bias

No evidence of publication bias was identified in OS in subgroups defined by high and low tumor EREG mRNA expression using a contour-enhanced funnel plot (Supplementary Figure 1) or Begg's test (Z = 0.25, *p*-value = 0.805).

## DISCUSSION

EGFR and its ligands play central roles in the development of epithelial tumors, including colorectal cancers [27]. However, the predictive value of AREG and EREG tumor mRNA levels is currently disputed. Most analyses focusing on AREG and EREG have been conducted in cohorts of patients who received cetuximab and did not include control patient groups that did not receive EGFR-targeting therapy [9, 15–24]. The present investigation was motivated by the lack of meta-analysis data regarding associations between expression of the EGFR ligands AREG and EREG and prognosis in mCRC.

This meta-analysis included nine cohorts: eight studies used anti-EGFR mAb therapy alone or in combination with chemotherapy, and one study used only chemotherapy. We found that high AREG and EREG mRNA expression in primary tumors was associated with prolonged OS in mCRC patients. High AREG and EREG mRNA expression was also associated with longer PFS. These results suggest that AREG and EREG expression are independent prognostic markers in mCRC patients, whether or not chemotherapy is accompanied by anti-EGFR mAb therapy.

We evaluated AREG and EREG mRNA expression in mCRC, whereas previous studies examined AREG and EREG protein levels [8, 28, 29]. Tissue and pretreatment serum levels of AREG and EREG protein were negatively correlated with clinicopathological characteristics, such as depth of tumor invasion, distant metastases, and nerve invasion. Thus, determining the levels of these ligands might help identify patients who require adjuvant treatment and intensive follow-up [8, 29]. However, AREG and EREG levels were also inversely proportional to total skin toxicity grades, suggesting the need for a new dose-modulation strategy for anti-EGFR antibodies [28]. Finally, Hobor and Loupakis reported increased levels of circulating EGFR ligands in mCRC patients treated with cetuximab and irinotecan at the time of disease progression, suggesting a potential role for these ligands in acquired resistance to drug treatment [12, 30, 31]. Here, we found specifically that ligand mRNA expression in the primary tumor was related to survival in mCRC patients.

Some studies have shown that ligand-induced EGFR activation results from autocrine or paracrine stimulation. EREG organizes epidermal structure by regulating keratinocyte proliferation and differentiation. A previous report revealed that EREG plays an autocrine role in the proliferation of human epithelial cells [32]. AREG is a major autocrine factor in human keratinocytes, and its expression is developmentally regulated in human skin epithelium and mesenchyme during morphogenesis [33]. Ligand binding triggers the homo- or heterodimerization of EGFR receptors, which then activates mitogenic and anti-apoptotic signaling cascades [34, 35]. AREG- and EREG-induced upregulation of EGFR is not only a key mediator of intestinal neoplastic transformation, but also a positive predictor of sensitivity to EGFR inhibition in CRC [36–39].

In this meta-analysis, AREG/EREG mRNA overexpression was associated with longer OS in RAS-WT patients who received cetuximab or panitumumab treatment. In addition, AREG overexpression was associated with longer PFS in RAS-WT patients. Therefore, AREG and EREG mRNA expression might be predictive biomarkers for the success of anti-EGFR therapy in RAS-WT patients, indicating the presence of ligand-driven autocrine oncogenic EGFR signaling [27, 28]. Blocking this AREG/EREG-induced activation of EGFR might promote cancer cell death. In contrast, this pathway might be redundant or irrelevant, perhaps due to a low level of activation by AREG and EREG, in cancer cells, thus rendering the blockade of EGFR by cetuximab ineffective. Furthermore, AREG and EREG did not have predictive value in patients with *KRAS* mutations, indicating that other oncogenic intracellular signaling pathways, including non-RAS-RAF-MAPK pathways, are activated in KRAS-MT mCRC [40].

Limitations that apply to meta-analysis studies in general, including differences in study populations, analytic techniques, and randomization, should be considered when interpreting these results. Additionally, AREG and EREG levels vary greatly among patients, and appropriate cutoff points for high *vs*. low expression should be investigated further using independent datasets. Prospective, randomized, and controlled studies using validated assays and optimized cutoff points will help clarify the value of AREG and EREG as predictive biomarkers in the clinical setting.

This study demonstrated for the first time the utility of high EREG and AREG mRNA expression within primary tumors as an independent favorable prognostic biomarker for mCRC patients. In addition, our results suggest that anti-EGFR mAb therapy might be particularly beneficial in RAS-WT patients with high AREG and EREG expression. These results suggest that further examination of these ligands in controlled trials is warranted.

## MATERIALS AND METHODS

### Search strategy and study selection

We searched the EMBASE, PubMed, and Cochrane Library databases for research published on or prior to March 15, 2016 using the following search terms (treated as Mesh terms or free text): (“colorectal cancer”, or “Colorectal Neoplasms” or “metastatic colorectal cancer” or “advanced colorectal cancer”) and (“Amphiregulin” or “AREG mRNA, human” or “Epiregulin” or “EREG mRNA, human”). We also searched the clinical trial registration website (ClinicalTrials.gov) to obtain information on registered clinical trials. This study is a meta-analysis and did not involve subjects; therefore, ethical approval was not required.

The following criteria were included for study selection: (1) patients were diagnosed with metastatic colorectal cancer according to pathological material and imaging; (2) mCRC patients were treated with standard therapy, either alone or in combination with anti-EGFR antibody; (3) studies had follow-up data on OS or PFS outcomes; (4) studies assessed hazard ratios associated with differences in AREG and/or EREG mRNA expression in mCRC patients; and (5) studies were prospective. Studies were excluded if they did not provide sufficient quantitative data regarding AREG and/or EREG expression status. Study selection was conducted according to the Preferred Reporting Items for Systematic Reviews and Meta-Analyses statement (Supplementary Material Appendix A1).

### Data extraction and quality assessment

Data extraction was conducted by two independent investigators (CJ and ZJ), with any discrepancies resolved by a third investigator (YHJ). For each study, the following information was extracted: year of publication, first author's name, treatment arm, trial phase, and *p*-value of HRs calculated between high and low AREG or EREG mRNA expression groups and OS or PFS.

The quality of all included trails was assessed using the Jadad scale, and scores ranged from 0 to 5, with a high score indicating a high-quality study [41].

### Date synthesis and statistical analysis

The primary summary measures were hazard ratio (HR) and 95% confidence interval (CI). HR and 95% CI were extracted from each study to calculate the overall HRs and 95% CIs. Heterogeneity among trials was assessed by using the Q statistic and I^2^ tests. Heterogeneity was considered statistically significant when P_heterogeneity_ < 0.1 or I^2^>40%. If heterogeneity existed, the data was analyzed using a random-effects model; if heterogeneity did not exist, a fixed-effects model was used [12, 42]. Statistical tests with *p*-values < 0.05 were considered significant. Contour-enhanced meta-analysis funnel plots and Begg's test were performed to assess publication bias [43, 44]. All statistical analyses were performed with R software, version 3.0.3 (The R Foundation for Statistical Computing,http://www.r-project.org).

## SUPPLEMENTARY MATERIALS


